# Pylore Krishnaier Rajagopalan: Pioneer in Kyasanur Forest Disease Research and His Contributions to Zoonotic Disease Studies

**DOI:** 10.7759/cureus.68831

**Published:** 2024-09-06

**Authors:** Pooja Mary Vaishali, Nisha Boopathy

**Affiliations:** 1 Department of Community Medicine, Saveetha Medical College and Hospital, Saveetha Institute of Medical and Technical Sciences, Saveetha University, Chennai, IND

**Keywords:** biographies, historical vignette, kyasanur forest disease, medical innovation, medical stories, pylore krishnaier rajagopalan

## Abstract

Pylore Krishnaier Rajagopalan was a distinguished public health expert who made significant breakthroughs in the research of Kyasanur forest disease (KFD), a prevalent tick-borne viral ailment in South India. His extensive research on KFD provided vital insights into the impact of the disease on public health and considerably advanced our knowledge of it. Rajagopalan's work also encompasses the study of zoonotic diseases, where he made substantial contributions to our understanding of their epidemiology and control. His research on KFD was instrumental in comprehending the disease transmission dynamics, clinical manifestations, and control strategies, resulting in improved management practices in regions where the disease is endemic. Rajagopalan's contributions have had a long-lasting impact on public health practices, as his work has influenced both scientific research and public health policies. The enduring effects of his work can be observed in the enhanced disease surveillance, outbreak response, and comprehension of zoonotic disease dynamics, which will continue to inform contemporary public health practices.

## Introduction and background

Pylore Krishnaier Rajagopalan was a highly respected Indian public health expert who made significant contributions to the study of Kyasanur forest disease (KFD) and other zoonotic diseases. Born in 1936, Rajagopalan pursued medical education at Madras Medical College, where he developed a strong interest in epidemiology and infectious diseases. Throughout his early career, he honed his skills in disease surveillance and control while working in various public health roles [1]. Rajagopalan's extensive fieldwork and research have focused on KFD, a disease prevalent in the Kyasanur forest region of Karnataka, India. His groundbreaking research provided critical insights into the epidemiology, transmission, and control of tick-borne viral diseases [2]. Prior to his work, KFD was poorly understood, and Rajagopalan’s efforts shed light on the complex interactions between wildlife, ticks, and humans. His findings have had a lasting impact on zoonotic disease management, influencing public health strategies and policies not only in India but also globally. Rajagopalan emphasized the importance of surveillance and early intervention in controlling zoonotic outbreaks, which has been critical for managing similar diseases worldwide [3].

This critique focuses on several key aspects of Rajagopalan's contributions: his research on KFD, a detailed examination of his role in identifying the disease, its transmission dynamics, and the development of control measures. It includes an in-depth discussion of his field studies and laboratory work, which provide foundational knowledge about KFD. The critique also addresses the implications for zoonotic disease management, analyzing how Rajagopalan's findings on KFD have informed broader strategies for managing zoonotic diseases. Additionally, it explores how his work has influenced disease surveillance, public health responses, and policy development. Finally, this section evaluates Rajagopalan's legacy and impact, highlighting his lasting influence on public health and the continued relevance of his research to other zoonotic diseases.

## Review

Pre-KFD conditions

Prior to the groundbreaking research conducted by Rajagopalan on zoonotic diseases, particularly in India, the field was not well developed, especially in KFD. The understanding of diseases transmitted from animals to humans, known as zoonotic diseases, is limited, and many such diseases are either unrecognized or poorly managed. In the early 20th century, there was a lack of comprehensive surveillance and diagnostic tools for these diseases, resulting in high mortality rates owing to misdiagnosis or attribution to other causes. For instance, prior to the discovery of KFD, the identification of pathogens transmitted by ticks was rudimentary, and many diseases with similar symptoms were either combined or misunderstood [4]. Moreover, the public health infrastructure in rural and forested areas is inadequate, further exacerbating the challenges of managing and controlling outbreaks.

Emergence of KFD

The outbreak of KFD in the early 1950s in the Kyasanur forest region of Karnataka, India, as an acute viral illness affecting both humans and animals, was a pivotal moment in understanding zoonotic diseases in the country. Initially presenting with symptoms similar to those of other febrile illnesses, KFD quickly spread and had high mortality rates, particularly in rural communities [5]. This prompted a closer examination of the disease, and research on KFD by Rajagopalan was initiated in the context of heightened concern. His work was instrumental in identifying the viral etiology of the disease and its transmission cycle involving ticks and wildlife, particularly the monkey reservoir. Prior to Rajagopalan's research, the disease was poorly understood, and effective public health responses were lacking. His investigations provided crucial insights into the epidemiology of the disease and led to the development of control measures that significantly reduced the impact of KFD [6].

Rajagopalan’s contributions

Rajagopalan played a pivotal role in recognizing and comprehending KFD, a viral illness transmitted by ticks. His research began in the early 1950s when KFD emerged as a significant health concern in the Kyasanur forest region of Karnataka, India [7]. He conducted field investigations and laboratory studies to identify the causative agent of the disease, which initially exhibited symptoms such as high fever, bleeding, and neurological issues in both humans and animals [8]. Rajagopalan's extensive fieldwork, including the collection of tick samples and monitoring of infected animals, has led to the isolation and identification of KFD virus. His research uncovered the disease transmission cycle, involving ticks and non-human primates as reservoirs. This study was instrumental in understanding the epidemiology of KFD and establishing its viral cause.

Rajagopalan's work on KFD has greatly advanced the study of zoonotic diseases. He has developed innovative methods for studying tick-borne viruses, such as improved techniques for isolating and characterizing viral pathogens. Additionally, his research illuminated the complex relationships between ticks, wildlife, and humans, contributing to a better understanding of the ecology of zoonotic diseases. As a result of his pioneering efforts, new diagnostic techniques and control strategies have been developed, including the implementation of tick control measures and vaccination campaigns [9]. Rajagopalan emphasized the importance of integrated disease management strategies, which have also been applied to other zoonotic diseases [10].

Rajagopalan's research on zoonotic disease control policies has had a considerable influence on the management of tick-borne diseases and KFD [11]. His work emphasized the necessity of effective surveillance and control measures, which subsequently led to the implementation of public health initiatives aimed at controlling KFD. Additionally, Rajagopalan's research has affected policy decisions related to vaccination, vector control, and public education, contributing to the development of comprehensive disease management strategies [12]. His work has set a precedent for addressing other zoonotic diseases with similar ecological and epidemiological characteristics, and his contributions have been vital in shaping global public health policies and practices related to zoonotic diseases.

Impact of Rajagopalan’s work

Rajagopalan's research on KFD had an immediate and significant impact on disease management in the affected regions. His discovery of KFD virus and elucidation of its transmission cycle have resulted in the implementation of targeted control measures. These measures included enhanced surveillance of tick populations, public awareness campaigns, and the development of specific interventions to manage outbreaks. Rajagopalan established local health initiatives to monitor and control the spread of KFD [13]. Vaccination campaigns have been launched to protect high-risk populations, and tick control programs have been implemented to reduce the incidence of the disease. In addition, Rajagopalan's research facilitated the development of diagnostic tools that allowed quicker identification and management of KFD cases. Rajagopalan's influence on zoonotic disease research and control went beyond the confines of KFD. His work has formed the basis for global advancements in disease monitoring and prevention. Methodologies developed for studying tick-borne viruses have been applied to other zoonotic diseases, resulting in enhanced diagnostic and surveillance techniques. Rajagopalan's research has had a lasting impact on global health strategies for zoonotic diseases [14]. He contributed to the creation of international guidelines for managing tick-borne diseases and emphasized the importance of an integrated approach to disease control that encompasses vector management, vaccination, and public health education. The wide-ranging impact of Rajagopalan's work is evident in ongoing research and advancements in the field of zoonotic diseases. His contributions have informed global health policies and have been instrumental in shaping strategies for monitoring and controlling emerging zoonotic threats.

Legacy of Rajagopalan

Rajagopalan's influential research on KFD had a dramatic impact on the realm of zoonotic disease research and public health. His work provides fundamental principles for the study of tick-borne viruses and zoonotic diseases. By elaborating on the epidemiology and transmission of KFD, Rajagopalan has significantly contributed to the development of new research methodologies and public health strategies [15]. His innovative techniques in disease surveillance and vector control have had lasting effects on subsequent research on zoonotic diseases. The models and frameworks derived from the KFD studies have been applied to other emerging zoonotic diseases, allowing for more efficient monitoring and control strategies worldwide. The emphasis of Rajagopalan on integrating field research with public health interventions has become a crucial component of zoonotic disease management.

Rajagopalan had a noteworthy impact on contemporary medical practices, particularly in disease surveillance, prevention, and management. His research has led to significant advancements in the understanding of the transmission of zoonotic diseases and shaped current practices for managing similar outbreaks. The principles derived from Rajagopalan's studies are evident in modern disease surveillance systems that incorporate integrated approaches to vector management, early detection, and rapid response. Rajagopalan's emphasis on the interdisciplinary nature of zoonotic disease research has influenced contemporary public health strategies, including the use of genomic and ecological data to predict and mitigate disease outbreaks. His contributions have also led to the development of new vaccines and diagnostic tools for zoonotic diseases, improving their capacity to respond to emerging threats. Rajagopalan's work has become a model for combining field research with practical public health applications, thereby influencing how modern medicine approaches the prevention and control of zoonotic diseases.

Accolades and recognition

Rajagopalan has been recognized for his substantial influence on zoonotic diseases. His pioneering research on KFD earned him a range of awards and acknowledgments within both the scientific and public health spheres. To commemorate his contributions, several research institutes have been established in his name, focusing on tropical and zoonotic diseases as well as vector-borne illnesses. Rajagopalan has received multiple honors posthumously, such as the "Global Health Pioneer Award," for his invaluable contributions to understanding and managing zoonotic diseases [16]. Finally, monuments and plaques have been erected in his honor at various research sites and institutions that acknowledge and commemorate their significant impact on public health.

## Conclusions

Rajagopalan's pioneering research on KFD has had a profound effect on the field of zoonotic diseases. Rajagopalan conducted an extensive investigation of KFD's epidemiology and transmission dynamics, establishing a critical foundation for understanding tick-borne viruses and zoonotic infections. His work has advanced scientific knowledge and informed public health strategies for disease control. Rajagopalan's innovative methods continue to be highly relevant today, particularly in light of emerging zoonotic threats to global health. His contributions have significantly influenced modern surveillance and prevention practices, highlighting their lasting impacts on global health security. This study's future implications are substantial, as it establishes a foundation for subsequent investigations into the monitoring and control of emerging zoonotic diseases, particularly in the context of climate change and ecosystem perturbations. Building upon Rajagopalan's seminal research, there exist opportunities to develop more sophisticated diagnostic methodologies and systems for the early detection of disease outbreaks. Furthermore, the integration of approaches from veterinary medicine, human healthcare, and environmental science could enhance efforts to prevent and manage zoonotic diseases, potentially leading to improved public health preparedness in the coming years.
